# Impacts of the 2011 Tsunami on Sediment Characteristics and Macrozoobenthic Assemblages in a Shallow Eutrophic Lagoon, Sendai Bay, Japan

**DOI:** 10.1371/journal.pone.0135125

**Published:** 2015-08-04

**Authors:** Gen Kanaya, Takao Suzuki, Eisuke Kikuchi

**Affiliations:** 1 National Institute for Environmental Studies, 16–2 Onogawa, Tsukuba 305–8506, Japan; 2 Biological Institute, Graduate School of Science, Tohoku University, Sendai 980–8578, Japan; 3 Graduate School of Life Sciences, Tohoku University, Aramaki-Aoba, Sendai 980–8578, Japan; 4 EEC, Miyagi University of Education, 149 Aramaki-Aoba, Sendai 980–0845, Japan; Centro de Investigacion Cientifica y Educacion Superior de Ensenada, MEXICO

## Abstract

A huge tsunami is one of the greatest disturbance events in coastal benthic communities, although the ecological consequences are not fully understood. Here we examined the tsunami-induced changes in the sediment environment and macrozoobenthic assemblage in a eutrophic brackish lagoon in eastern Japan. The 7.2-m-high tsunami completely replaced muddy sediment with drifting sea sand throughout the lagoon, leading to the drastic changes in quantity and quality of sedimental organic matters, sulfide contents, and sediment redox condition. Intensive physical stress devastated the benthic community, but the disappearance of sulfidic muddy bottoms significantly improved the habitat quality for macrozoobenthos. The re-established macrozoobenthic community after 5 months was characterized by (1) a 2-fold higher total density, but sharp declines in species richness, diversity, and evenness; (2) an increased density of opportunistic taxa (e.g., polychaete *Pseudopolydora* spp. and amphipod *Monocorophium uenoi*) in newly created sandy bottoms; and (3) disappearance of several dominant taxa including bivalves and chironomid larvae. These findings indicate that the sensitivity and recovery potential of macrozoobenthos were highly taxa-specific, which was closely related to the taxa’s ecological characteristics, including tolerance to physical disturbance, life-history traits, and life form. Our data revealed the rapid recolonization of opportunistic macrozoobenthos after a huge tsunami, which would contribute to the functional recovery of estuarine soft-bottom habitats shortly after a disturbance event.

## Introduction

In natural ecosystems, equilibrium in the biotic community is not usually attained because severe disturbance events occur with various intensities and frequencies [1]. Disturbance often induces changes in the structure and function of ecosystems, including those in coastal soft-bottom habitats [2, 3]. Generally, it takes several years to recover the community structure after an intense disturbance event because each species has different life-history traits and recolonization rates [4–6]. The impacts of disturbance and the following recovery process are also highly site- and context-specific [5, 7, 8]. Thus, it has been a major focus of marine community ecology to understand the recolonization and succession patterns after disturbance events.

A huge tsunami is one of the most intensive physical disturbance events in shallow coastal ecosystems. Tsunamis modify the habitat structure through destruction of coastal structures, massive sediment scouring, and sedimentation of tsunami deposits [6, 9, 10]. This often leads to a loss or modification of associated biota in the benthic community [6, 10, 11]. However, the ecological consequences of huge tsunamis are not well understood because these extreme events rarely occur [12]. To our knowledge, tsunami impacts on the sediment physico-chemical parameters (e.g., redox condition, organic contents, and granulometry) and the resulting consequences on the associated benthic community have not been examined in intertidal soft-bottom habitats. Only a few studies have reported on the effects of a tsunami on the community structure and local population of macrozoobenthos on tidal flats [10, 11, 13]. The lack of pre-tsunami data sets has also made it difficult to conduct quantitative assessments of tsunami impacts on sediment environments and benthic communities in intertidal flats.

A megathrust earthquake (M 9.0) occurred on 11 March 2011 at the plate boundary (38.322°N, 142.369°E) in the western Pacific region off the eastern coast of Japan [12]. The earthquake induced a huge tsunami (up to 20 m in maximum inundation depth) that heavily damaged the Pacific coast of Japan, including the coastal areas along Sendai Bay [14]. The tsunami caused massive physical disturbances in tidal flats and salt marshes [15], leading to a loss of species diversity, the destruction of local populations, and/or an increase in specific taxa in the benthic community [11, 13, 16]. Macrozoobenthos play a key role in biogeochemical cycles in marine soft-bottom communities through their feeding and bioturbation activities [2]. Therefore, tsunami-induced changes in macrozoobenthic community structure, especially the disappearance of specific functional groups [6, 11, 13], should lead to a loss of its ecosystem function at a broader spatial scale.

Gamo Lagoon (38.257°N, 141.014°E) is a shallow hypertrophic lagoon located on the northern side of the Nanakita River estuary, Sendai Bay. On 11 March 2011, the lagoon was hit by a tsunami with a 7.2-m inundation depth. The tsunami drastically altered the topography, vegetation, and faunal composition in this lagoon [16, 17]. The sand bar was broken, and the reed marsh and coastal forests and sand dune vegetation mostly disappeared. In our previous reports, alteration of macroinvertebrate diversity had been assessed only from the qualitative survey data [16, 17]. Prior to March 2011, yearly quantitative surveys had been conducted in this lagoon, covering the entire lagoon surface [18]. These data sets allow us to conduct quantitative and statistical analyses about the degree of disturbance caused by the huge tsunami on the environment and associated biota in the lagoon.

In this study, we examined sediment physico-chemical characteristics and macrozoobenthic abundance at 5 months after the tsunami using the same sampling protocol as used in the pre-tsunami surveys [19]. Sediment parameters including granulometry, total organic carbon (TOC), total nitrogen (TN), C/N ratio, stable carbon and nitrogen isotope ratios (*δ* ^13^C and *δ* ^15^N), redox potential (Eh), and sulfide contents (acid-volatile inorganic sulfide and free H_2_S) were measured. Sulfides, Eh value, TOC, and TN were measured as indicators of organic enrichment and sediment deterioration, while C/N, *δ* ^13^C, and *δ* ^15^N were used as indicators for origin of sediment organic matters [19]. Interannual changes in macrozoobenthic biodiversity, population density, and community structure were also assessed based on the data collected at 30 fixed stations in 2005, 2007, 2008, and 2011. In this study, we aimed to reveal (1) tsunami-induced changes in sediment physico-chemical properties; (2) species-specific responses to the tsunami disturbance; and (3) characteristics of the re-established macrozoobenthic community structure shortly after the huge tsunami event.

## Materials and Methods

### Study site description and ethics statements

**Fig 1:**
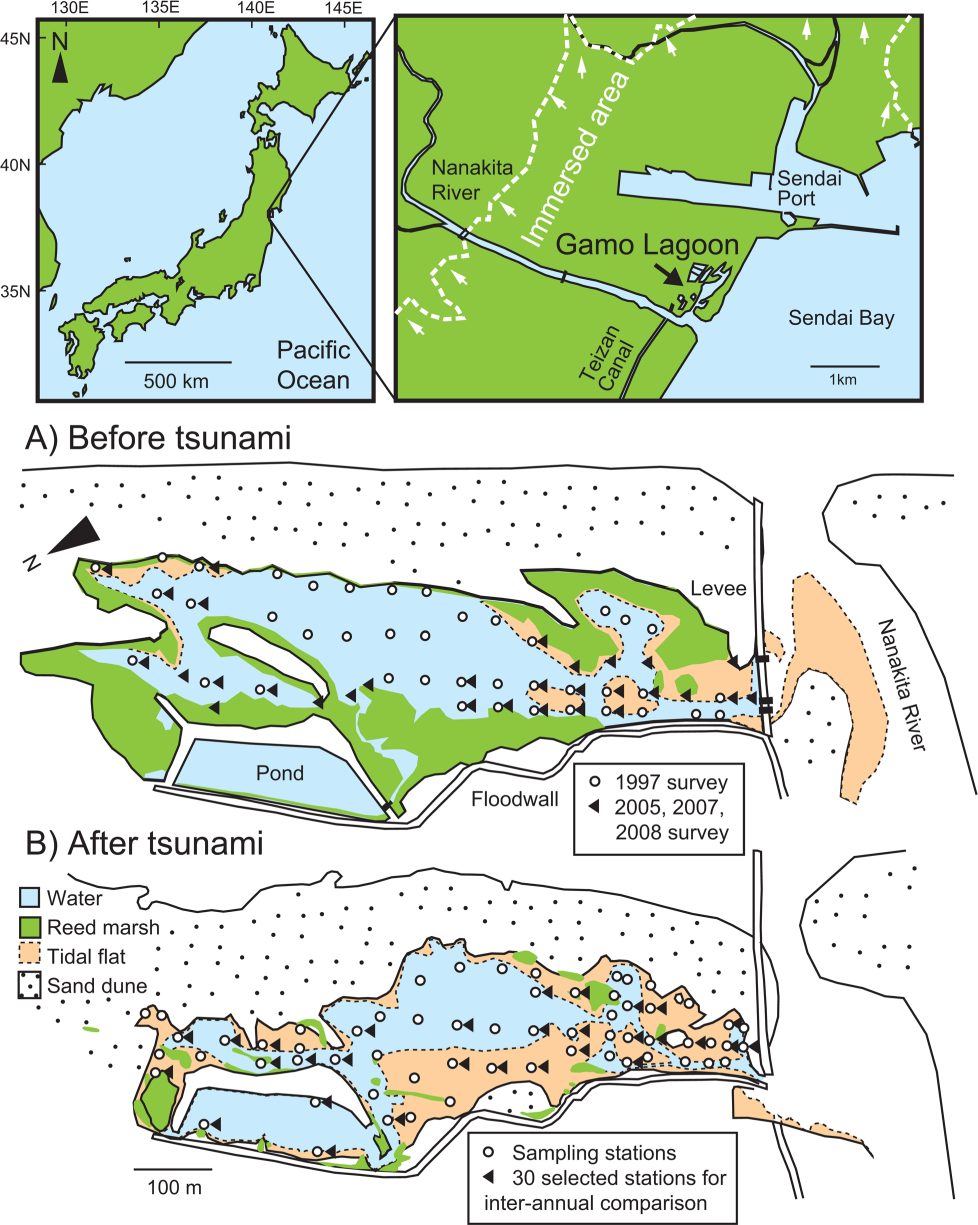
Locations of the sampling stations in Gamo Lagoon A) before and B) after the 2011 tsunami. Sediment characteristics were measured at 41 stations in 1997 and 63 stations in 2011 (white circles). Macrozoobenthos data at 30 stations in 2005 to 2008 (black triangles in A)) and in 2011 (black triangles in B)) were used for interannual comparison.

Gamo Lagoon (0.11 km^2^, mean water depth 0.8 m) is located on the northern side of the Nanakita River mouth, facing Sendai Bay, Japan (Fig 1). The lagoon is separated from the estuary by a stone levee with three water gates. The spring tidal range was about 0.5m. Salinity fluctuates tidally from less than 10 to over 25 [18]. Before the tsunami, the lagoon was highly eutrophic, characterized by high phytoplanktonic biomass (chl. *a* > 100 μg L^–1^) and the accumulation of organically enriched mud in the stagnant inner part [19, 20]. Sediment became muddy and sulfide-rich in the inner portion [19, 21]. A reed marsh consisting of *Phragmites australis* developed in the upper tidal zone. For field survey, no special permits were required to perform field surveys since the lagoon was not privately owned or legislatively protected. We did not involve protected species for this study.

### Tsunami impacts

On 11 March 2011, a huge tsunami drastically disturbed the lagoon and adjacent areas (Fig 1). The sand bar was completely destroyed, and sea water entered the lagoon directly for several months [15]. Most of the reed marsh, sand dune vegetation, and macroalgae disappeared [16]. After 2 months, the sand bar was reformed through deposition of drifting sea sand, leading to the re-establishment of the semi-enclosed lagoon shape. The disappearance of macrozoobenthic species, especially bivalves, gastropods, and decapods, and the temporary immigration of stenohaline marine species were noted after the tsunami [16].

### Sediment physico-chemical characteristics

Sediment parameters were measured at 41 and 63 stations in August 1997 and July 2011, respectively (Fig 1). The 1997 data had partly been reported in our previous report [19]. Eh was measured in the field at 5-cm depth using a hand-held ORP meter (RM-12P and RM-21P, TOA DKK). A sediment core (5 cm φ × 20 cm length) was collected from each station. Each core was sealed with rubber caps to keep it in an undisturbed condition and brought to the laboratory. The top 1-cm layer of the core was acidified with 1 M HCl and dried at 70°C for 48 h for TOC, TN, *δ* ^13^C, and *δ* ^15^N analyses. The sample was placed into a tin cup, combusted in an elemental analyzer, and introduced to a mass spectrometer (NC-2500 and DELTA Plus, Finnigan Mat). The C/N molar ratio was calculated from the contents. Isotope ratios are expressed in delta notation:

$$\delta^{13}C\;or\delta^{15}N\;\left(‰\right)=\left(R_{sample}/R_{standard}–1\right)\times 1000$$

where *R* is the ^13^C/^12^C or ^15^N/^14^N ratio for *δ* ^13^C or *δ* ^15^N, respectively. Pee Dee belemnite and atmospheric N_2_ were used as references for *δ* ^13^C and *δ* ^15^N, respectively. d-alanine and l-histidine were used as working standards.

A 30-ml aliquot was sampled from a 1 to 3 cm deep layer of the core using a small syringe corer (φ 2.8 cm). Silt-clay content (<0.063-mm mesh) of the sediment was determined by wet sieving after wet and dry weights (dried at 70°C for 48 h) were measured. A 10-ml aliquot was taken from a 4 to 5 cm deep layer for sulfide content analyses. First, free H_2_S was extracted by purging with pure N_2_ gas and absorbed into a 2.5% zinc acetate solution. Next, acid-volatile insoluble sulfide (AViS) was liberated as H_2_S under acidic conditions. Sulfide content in the solution was determined by iodometry.

### Macrozoobenthos

Macrozoobenthos was sampled at 41 stations in August 1997; 30 stations in June 2005, June 2007, and July 2008; and 63 stations in July 2011. At each station, nine sediment cores (i.e., 176.7 cm^2^, 30-cm depth) were collected using a PVC corer (5 cm φ × 80 cm length). The sediment was sieved *in situ* through a 1-mm mesh sieve, and the retained fraction was fixed in 5% neutralized formalin. In the laboratory, animals were sorted, transferred into 70% ethanol, and identified under a dissecting stereomicroscope. The entire data sets from 1997 and 2011 (41 and 63 stations, respectively) were used to compare the spatial distribution pattern of macrozoobenthos between the years. Data collected at 30 stations in 2005, 2007, 2008, and 2011 (see Fig 1) were used for interannual comparison of population density and community structure.

### Statistical analyses

Lagoonal mean values of each sediment parameter in 1997 and 2011 were compared by Welch’s *t*-test. A principal component analysis (PCA) was used to assess the tsunami-induced changes in sediment characteristics using the data sets from 1997 and 2011. A correlation matrix was calculated from nine normalized sediment parameters. Samples were plotted in a two-dimensional PCA plot along coordinates defined by two PC axes. Eigenvectors were inserted into the plot as arrows. One-way analysis of variance (ANOVA) and a Tukey–Kramer test were used to examine interannual changes in lagoonal mean macrozoobenthic density, Shannon’s diversity index (*H*ʹ), and species evenness (*J*ʹ). Homogeneity was tested by the Bartlett test, and, if necessary, data were log or square-root transformed. If it was the case that homogeneity of variance could not be achieved even after data transformation, we used the nonparametric Steel–Dwass test. Nonmetric multidimensional scaling (nMDS) was used to assess the inter-annual changes in macrozoobenthic community structure among 2005, 2007, 2008, and 2011 based on the Bray–Curtis similarity calculated from the square-root-transformed density. One-way analysis of similarity (ANOSIM) tested inter-annual difference in community structure. SIMPER procedures identified the discriminant species contributing most to the intergroup dissimilarity until the cumulative percentage achieved 60%. All multivariate analyses were performed using PRIMER ver. 6.2 software [22].

## Results

### Sediment characteristics

**Fig 2:**
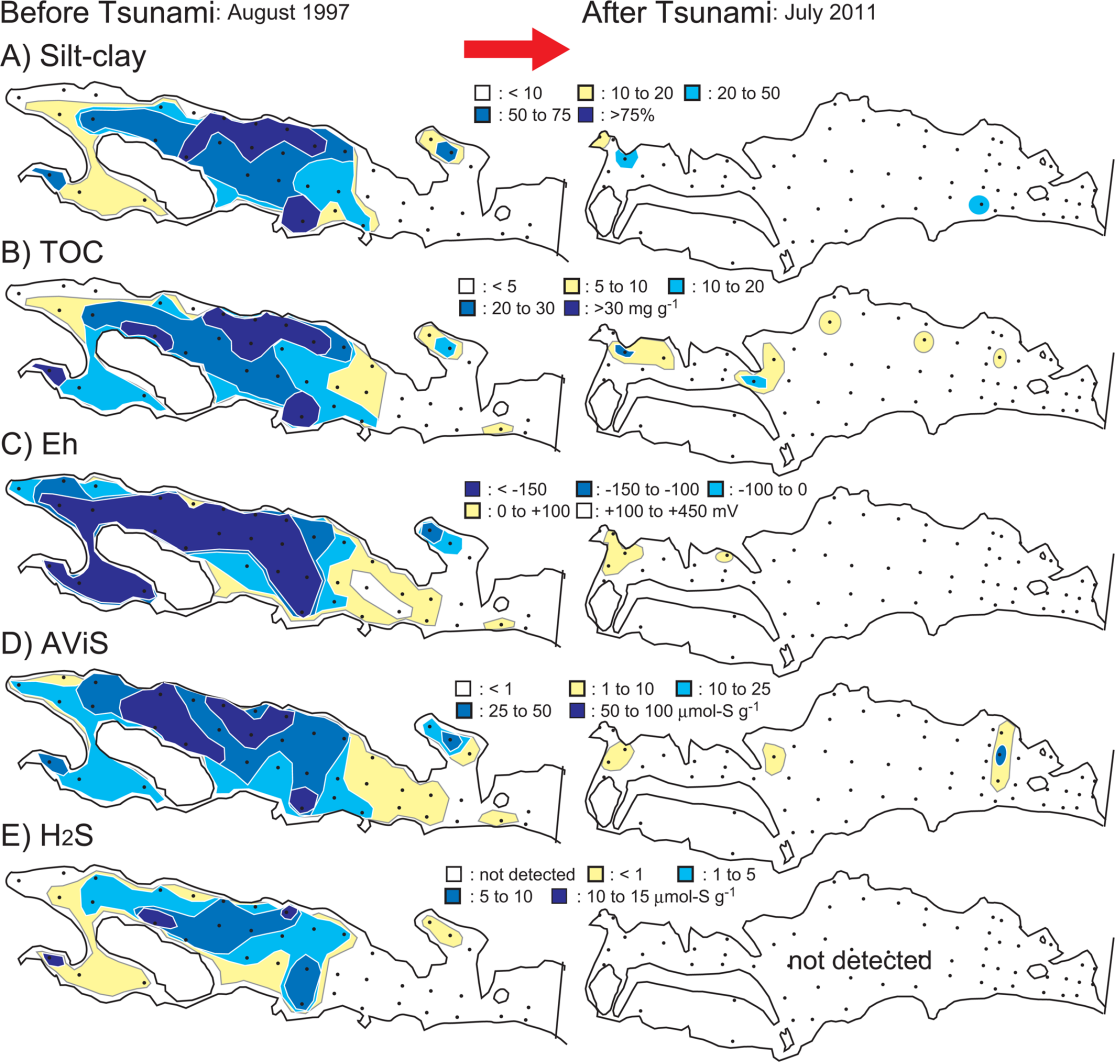
Spatial distribution of sediment properties in 1997 (left) and 2011 (right). A) Silt-clay, B) TOC, C) Eh, D) AViS, and E) H_2_S. Results for 1997 were modified from [19] and silt-clay data in 2011 was modified from [17].

The tsunami caused drastic changes in the sediment environment in Gamo Lagoon (Fig 2). Organic-rich sulfidic mud was mostly flushed away, and the sediment became sandy throughout the lagoon. Only a few patches of mud remained after the tsunami. Lagoonal averaged contents of silt-clay, TN, TOC, AViS, and H_2_S decreased significantly, concurrent with a sharp increase in sediment Eh values (Welch’s *t*-test, *p* < 0.001; Table 1). The *δ* ^13^C value of sediment organic matter (SOM) increased by 1.6‰ after the tsunami (*p* < 0.001), whereas the C/N ratio and *δ* ^15^N did not change (*p* > 0.05).

**Table 1:** Changes in sediment biogeochemical parameters in Gamo Lagoon.

Variables	1997	2011	Welch’s *t*-test	
	*n* = 41	*n* = 63	df	*t*-value
Silt-clay (%)	33.2 (31.9)	4.1 (4.6)	41.1	−5.81***
TN (mg g^–1^)	1.6 (1.3)	0.3 (0.3)	42.1	−6.23***
TOC (mg g^–1^)	14.7 (11.9)	2.6 (3.8)	45.5	−6.26***
C/N	11.1 (1.2)	10.3 (2.7)	82.8	−1.91 ^n.s.^
*δ* ^15^N (‰)	7.3 (1.3)	7.1 (1.8)	95.9	−0.43 ^n.s.^
*δ* ^13^C (‰)	−22.7 (1.3)	−21.1 (2.4)	97.7	4.44***
Eh (mV)	−51 (146)	296 (87)	59.1	13.7***
AViS (μmol g^–1^)	28.6 (27.7)	1.0 (4.8)	41.6	−6.33***
H_2_S (μmol g^–1^)	2.4 (3.6)	0 (n.d.)	40.0	−4.27***

Lagoonal mean value (SD) is shown for each parameter. TN: total nitrogen; TOC: total organic carbon; *δ* ^15^N: stable nitrogen isotope ratio; *δ* ^13^C: stable carbon isotope ratio; Eh: redox potential; AViS: acid-volatile insoluble sulfide. *n* = 57 for TN, C/N, and *δ* ^15^N in 2011 because six samples were below the detection limit.

***p < 0.0001; n.s.: not significant (*p* > 0.05), n.d.; not detected.

**Fig 3:**
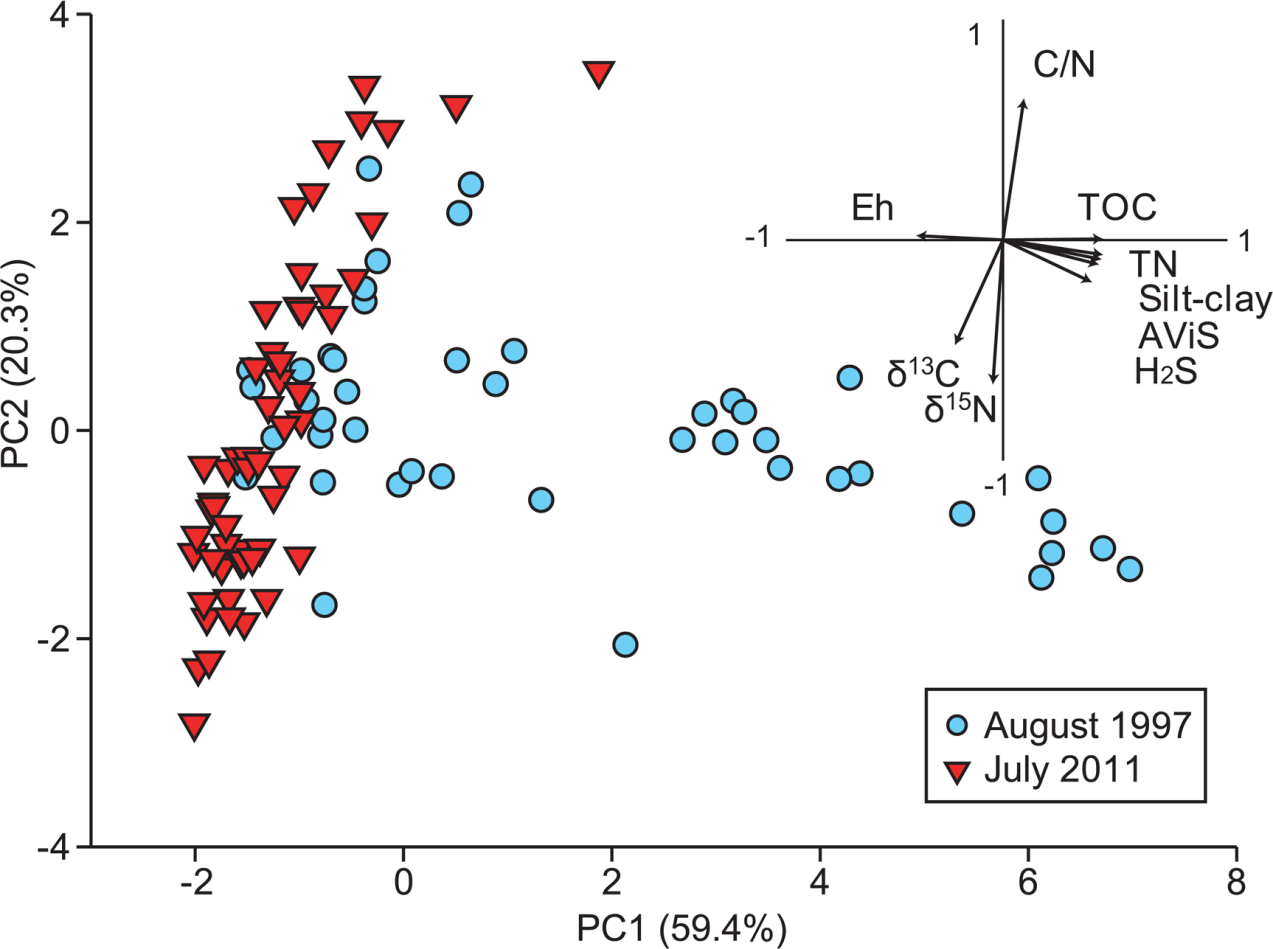
PCA plot based on nine normalized sediment variables in 1997 (*n* = 41) and 2011 (*n* = 63). The first two principal components (PC1 and PC2) accounted for 59.4% and 20.3% of total variance, respectively. Arrows indicate correlations between environmental variables and each PC axis (see Table 2).

The PCA plot clearly demonstrated the tsunami-induced changes in sediment characteristics (Fig 3). The first two PC axes explained 79.7% of the total variance (Table 2); PC1 was positively correlated with silt-clay, TN, TOC, H_2_S, and AViS but negatively with Eh (*p* < 0.001). A weaker negative correlation was detected with *δ* ^13^C (*p* < 0.01). This axis likely represents the degree of organic enrichment, chiefly characterized by sulfide accumulation and low Eh value (i.e., a highly reduced condition). PC2 was positively correlated with C/N ratio but was negatively correlated with *δ* ^13^C and *δ* ^15^N (*p* < 0.001). It likely reflects the changes in composition of SOM. In the plot, samples in 1997 were located broadly along both PC axes, whereas intersample variation along PC1 was far reduced in 2011.

**Table 2:** Eigenvalues, cumulative percent variation (Cum. %), and eigenvectors of a PCA examining sediment variables.

	Principal components		
	PC1	PC2	PC3
Eigenvalues	5.35	1.82	0.57
Cumulative %	59.4	79.7	86.0
Eigenvectors			
Silt-clay	0.413***	−0.081	0.076
TN	0.419***	−0.062	0.056
TOC	0.419***	0.005	0.083
C/N	0.090	0.606***	0.271**
*δ* ^15^N	−0.043	−0.619***	−0.400***
*δ* ^13^C	−0.202**	−0.444***	0.823***
Eh	−0.358***	0.018	0.185
AViS	0.401***	−0.103	0.088
H_2_S	0.372***	−0.177	0.178

Data were obtained at 41 and 63 stations in 1997 and 2011, respectively. TN: total nitrogen; TOC: total organic carbon; *δ* ^15^N: stable nitrogen isotope ratio; *δ* ^13^C: stable carbon isotope ratio; Eh: redox potential; AViS: acid-volatile insoluble sulfide.

**p < 0.01

***p < 0.001

### Spatial distribution of macrozoobenthos

**Fig 4:**
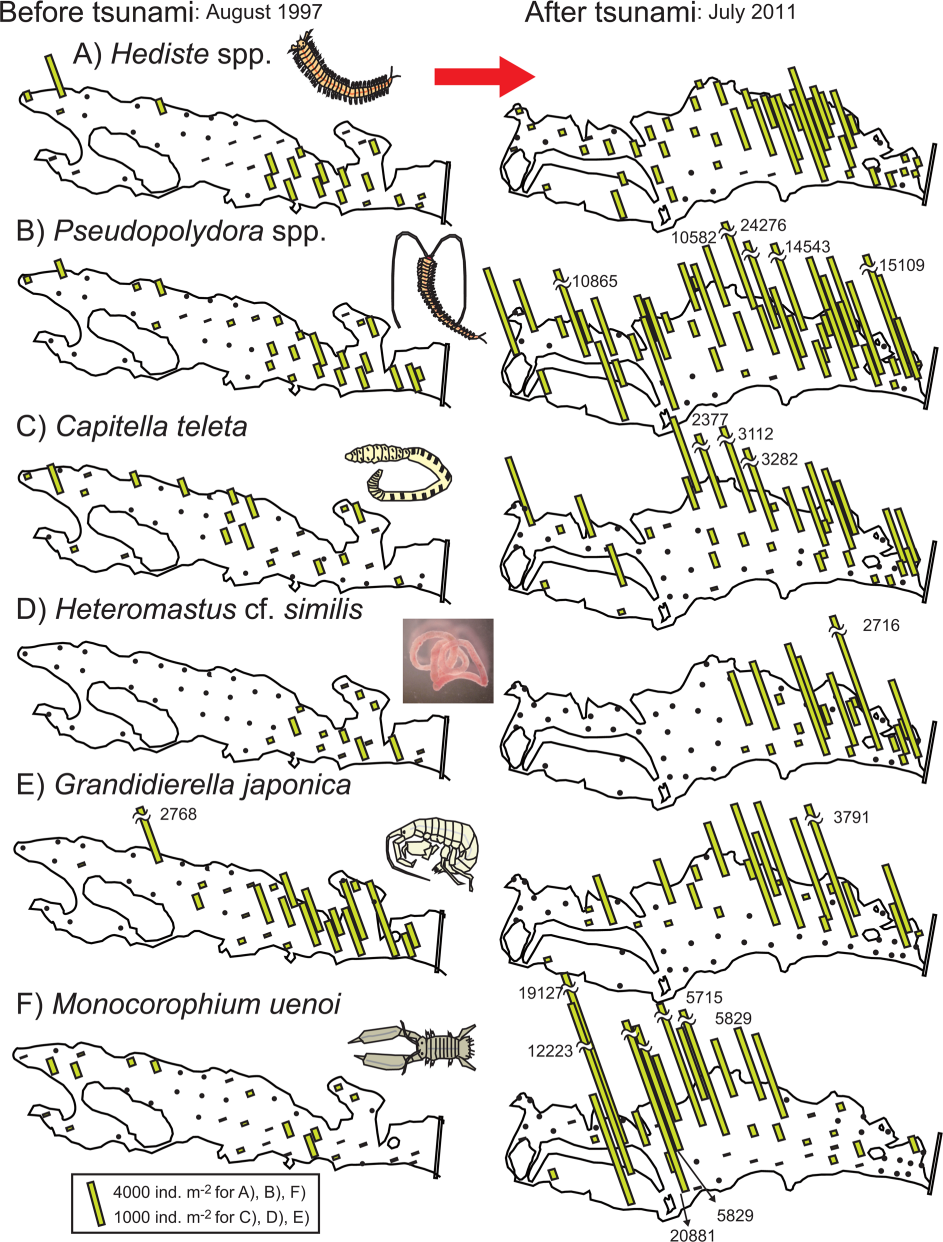
Distribution of 6 dominant polychaete and amphipod taxa in 1997 (left) and 2011 (right). A) *Hediste* spp. (*H*. *atoka* and *H*. *diadroma*), B) *Pseudopolydora* spp. (*P*. cf. *kempi* and *P*. *reticulata*), C) *Capitella teleta*, D) *Heteromastus* cf. *similis*, E) *Grandidierella japonica*, and F) *Monocorophium uenoi*. Each column indicates density (ind. m^–2^); note that the scale varies among the plots. Results for 1997 were partly reported in [19].

The distribution patterns of dominant polychaetes and amphipods differed distinctively between 1997 and 2011 (Fig 4). Overall, their density rose sharply at many of the sampling sites in 2011, especially those in the inner lagoon. Specifically, in 2011 the nereidid polychaetes *Hediste* spp. (consisting of *H*. *atoka* and *H*. *diadroma*), spionid polychaetes *Pseudopolydora* spp., and tube-dwelling amphipod *Monocorophium uenoi* densely colonized the inner lagoon, where they were rarely present in 1997. The capitellid polychaete *Capitella teleta* dominated muddy habitats (mostly in the inner lagoon) in 1997, whereas this species also occurred densely in sandy habitats near the lagoon mouth in 2011. In contrast, the distribution patterns of the capitellid polychaete *Heteromastus* cf. *similis* and the amphipod *Grandidierella japonica* did not apparently change between the two years; they mainly occurred near the lagoon mouth.

**Fig 5:**
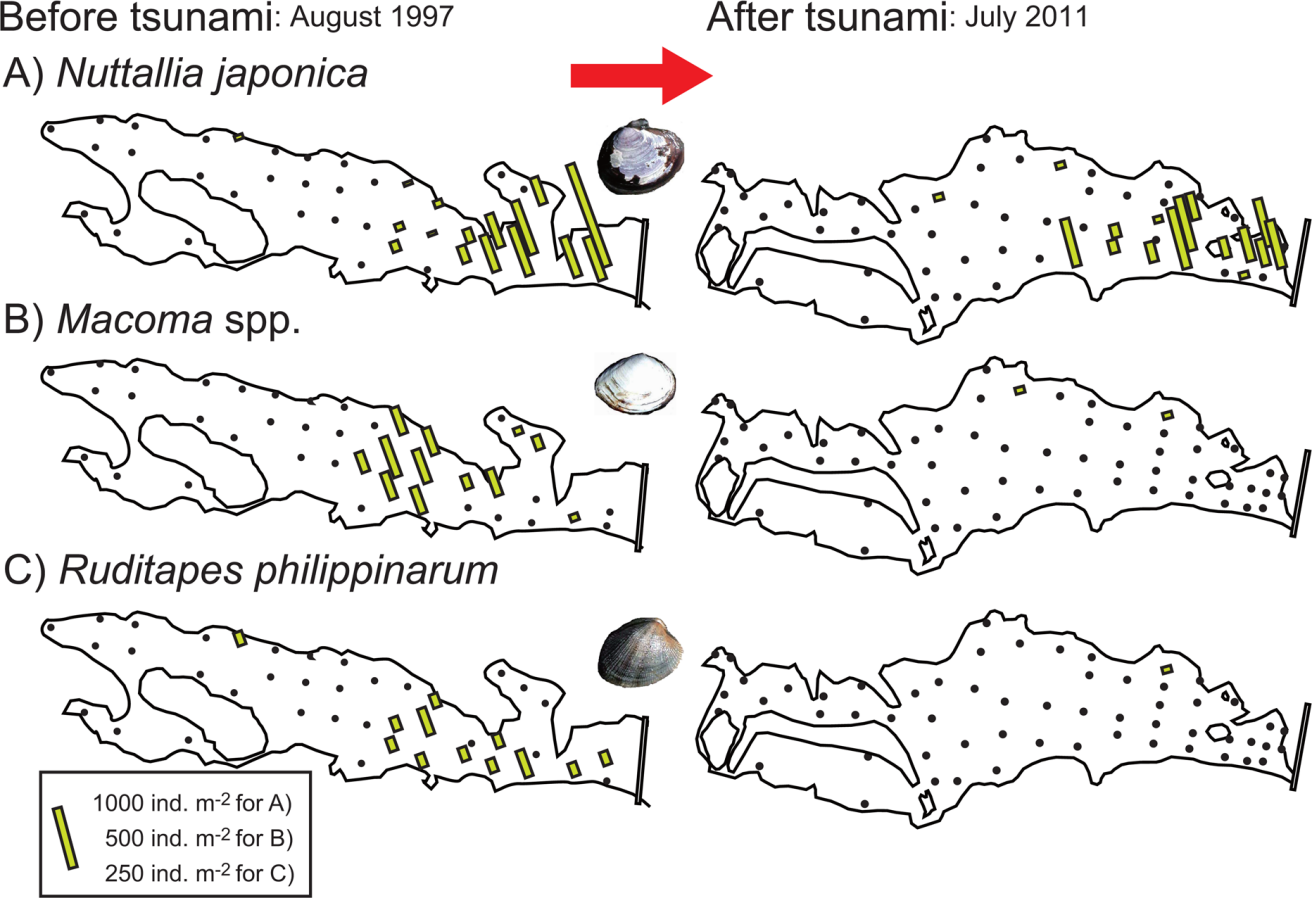
Distribution of 3 dominant bivalve taxa in 1997 (left) and 2011 (right). A) *Nuttallia japonica*, B) *Macoma* spp. (*M*. *contabulata* and *M*. *incongrua*), and C) *Ruditapes philippinarum*. The column length indicates density (ind. m^–2^); note that the scale varies among the plots. Results for 1997 were reported in [19].

In 1997, the dominant bivalve taxa, including *Nuttallia japonica*, *Macoma* spp. (mostly *M*. *contabulata* but also *M*. *incongrua*), and *Ruditapes philippinarum*, mainly occurred in the sandy to muddy sand habitats near the lagoon mouth (Fig 5). *N*. *japonica* mainly occurred on sandy flats in the vicinity of the levee, where the sediment was exposed to high tidal currents, whereas *Macoma* spp. mainly occurred in the central lagoon, where the sediment was rather muddy. *Ruditapes philippinarum* inhabited both of these areas. In 2011, only *N*. *japonica* maintained a population in the lagoon, whereas the other species had mostly disappeared. Only three individuals were collected in total for *Macoma* spp. and *R*. *philippinarum*. Most of the individuals of *N*. *japonica* seemed to be >1 year old, because the shell length was larger than 30 mm for 49 individuals and 10–30 mm for 21 individuals (*n* = 75 in total catch), as judged by the species’ reported growth rate [23].

### Changes in species diversity and population size

**Fig 6:**
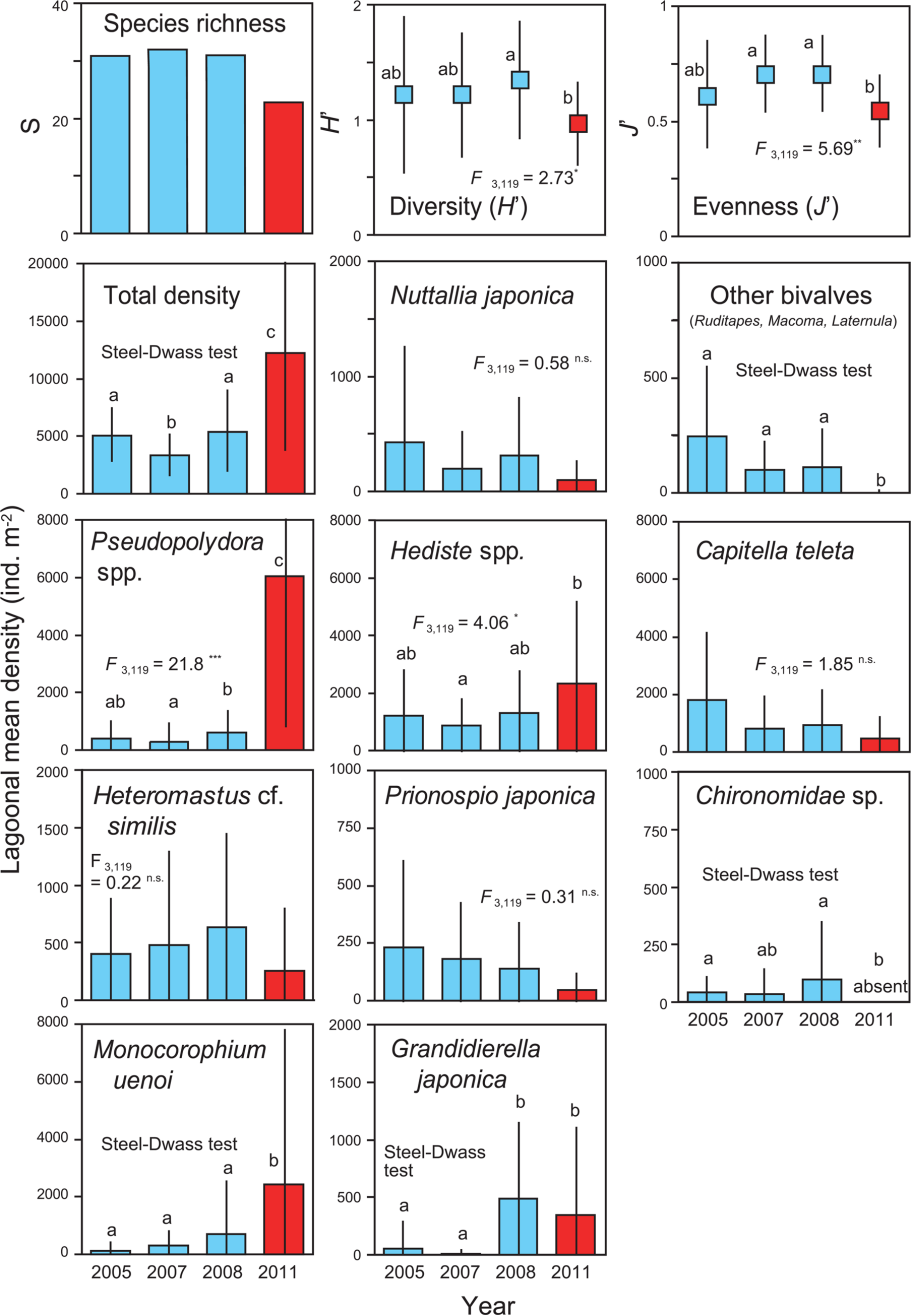
Interannual changes in species richness, *H*ʹ, *J*ʹ, and lagoonal mean density of total macrozoobenthos and 10 dominant taxa. The *F*-value for one-way ANOVA is shown with the significance level (**p < 0.01; ***p < 0.001; n.s., not significant). Different letters indicate significant differences among years (Tukey–Kramer test or Steel–Dwass test, *p* < 0.05). Bars represent SD (*n* = 30).

Species richness, *H*ʹ, *J*ʹ, lagoonal mean density of total macrozoobenthos, and population density of the 10 dominant taxa in 2005, 2007, 2008, and 2011 are shown in Fig 6. At the 30 stations, species richness sharply declined from those in 2005 to 2008 (31 or 32 taxa) to 23 taxa in 2011. *H*ʹ and *J*ʹ exhibited significant interannual variation (one-way ANOVA, *p* < 0.05), and 2011 values were significantly lower than those in 2008 (Tukey–Kramer test, *p* < 0.05). On the other hand, total macrozoobenthos rose sharply in 2011 and was about 2-fold higher than numbers in 2005 to 2008 (Steel–Dwass test, *p* < 0.05). This was mainly owing to the increased densities of polychaete *Pseudopolydora* spp. and amphipod *M*. *uenoi* in 2011.

Significant interannual changes in population density were detected for several infaunal taxa, including bivalves other than *N*. *japonica* (i.e., sum of *R*. *philippinarum*, *Macoma* spp., and *Laternula marilina*), the polychaetes *Pseudopolydora* spp. and *Hediste* spp., the insect Chironomidae sp. (larvae), and the amphipods *M*. *uenoi* and *G*. *japonica* (one-way ANOVA or Steel–Dwass test, *p* < 0.05; see Fig 6). The density of *Pseudopolydora* spp. and *M*. *uenoi* increased sharply in 2011 (Tukey–Kramer or Steel–Dwass test, *p* < 0.05), whereas Chironomidae sp. and all bivalves, except *N*. *japonica*, sharply declined or disappeared (*p* < 0.05). In contrast, no significant interannual changes were detected for the bivalve *N*. *japonica* and the polychaetes *C*. *teleta*, *H*. cf. *similis*, and *Prionospio japonica* (*p* > 0.05), although their population sizes slightly declined in 2011.

### Macrozoobenthic community structure

**Fig 7:**
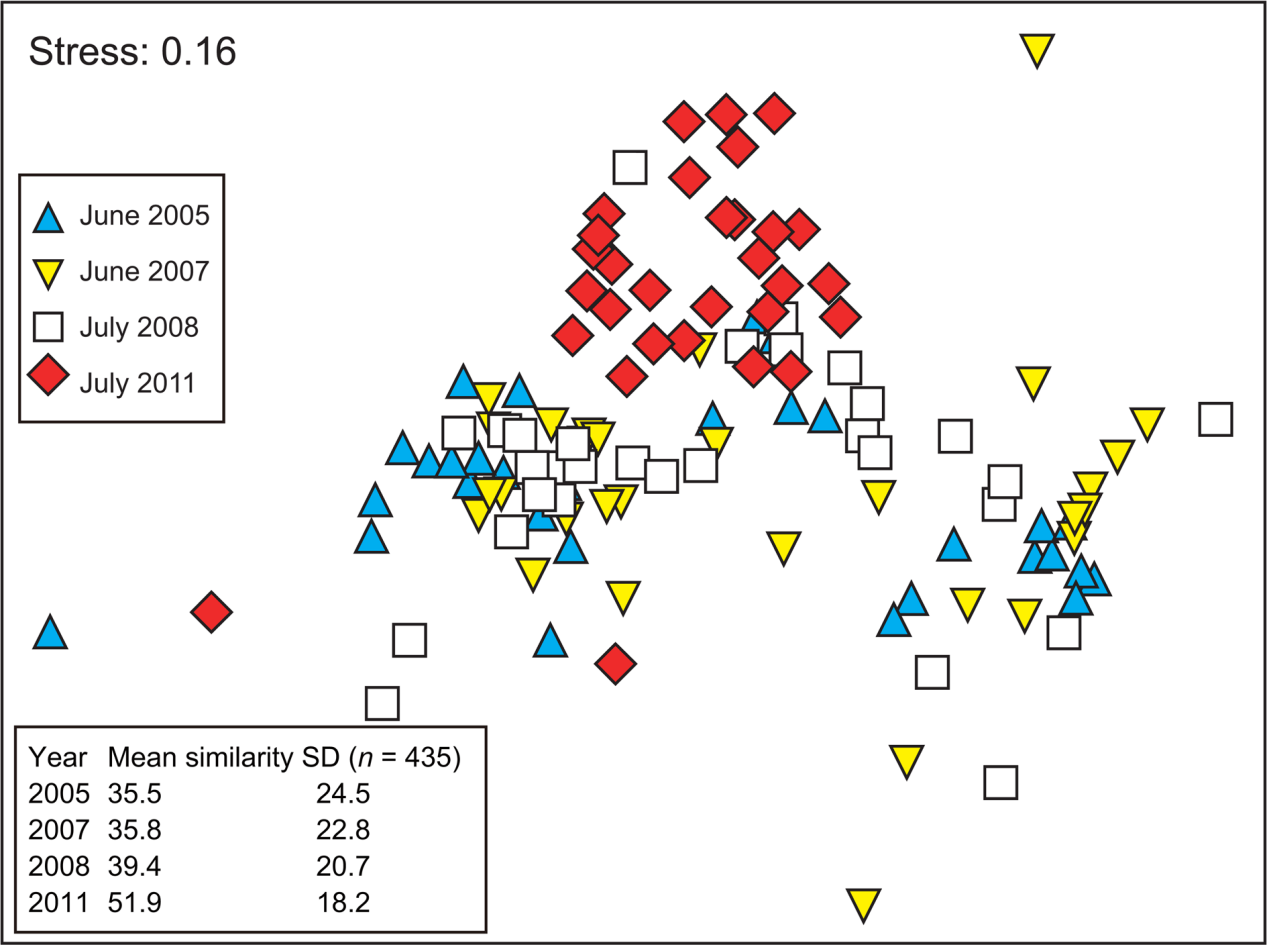
nMDS plot showing the interannual changes in macrozoobenthic community structure at 30 stations in 2005, 2007, 2008, and 2011. Bray–Curtis similarity was calculated from the square-root-transformed density. One-way ANOSIM detected significant interannual changes in community structure (global *R* = 0.191. *p* < 0.001). Mean intersample similarity was calculated for each year.

The nMDS plot showed inter- and intra-annual variations in macrozoobenthic community structure among 2005, 2007, 2008, and 2011 at the 30 sampling stations (Fig 7). Samples in 2011 were plotted around the upper-central part of the plot, whereas samples from other years were distributed in the lower portion. Samples in 2011 showed a more convergent distribution (i.e., higher intersample similarity) compared to those from other years. Averaged intersample similarity was much higher in 2011 (51.9 ± 18.2%, *n* = 435) than those before the tsunami (35.5 to 39.4%, *n* = 435 for each year). One-way ANOSIM detected significant changes in the community structure among years (global *R* = 0.191, *p* < 0.001), and a pairwise test demonstrated significant differences between 2011 and the other years with notably higher R values (*R* = 0.358 to 0.367, *p* < 0.001). Generally, R value is a measure of effect size among groups, ranging from 0 (no difference) to 1 (complete separation) [22]. Although significant differences were also detected for 2005 vs. 2008 and 2007 vs. 2008 (*R* = 0.052 to 0.065, *p* < 0.05), the R values were inconsequentially small. No significant difference was detected for 2005 vs. 2007 (*R* = 0.018, *p* > 0.05).

**Fig 8:**
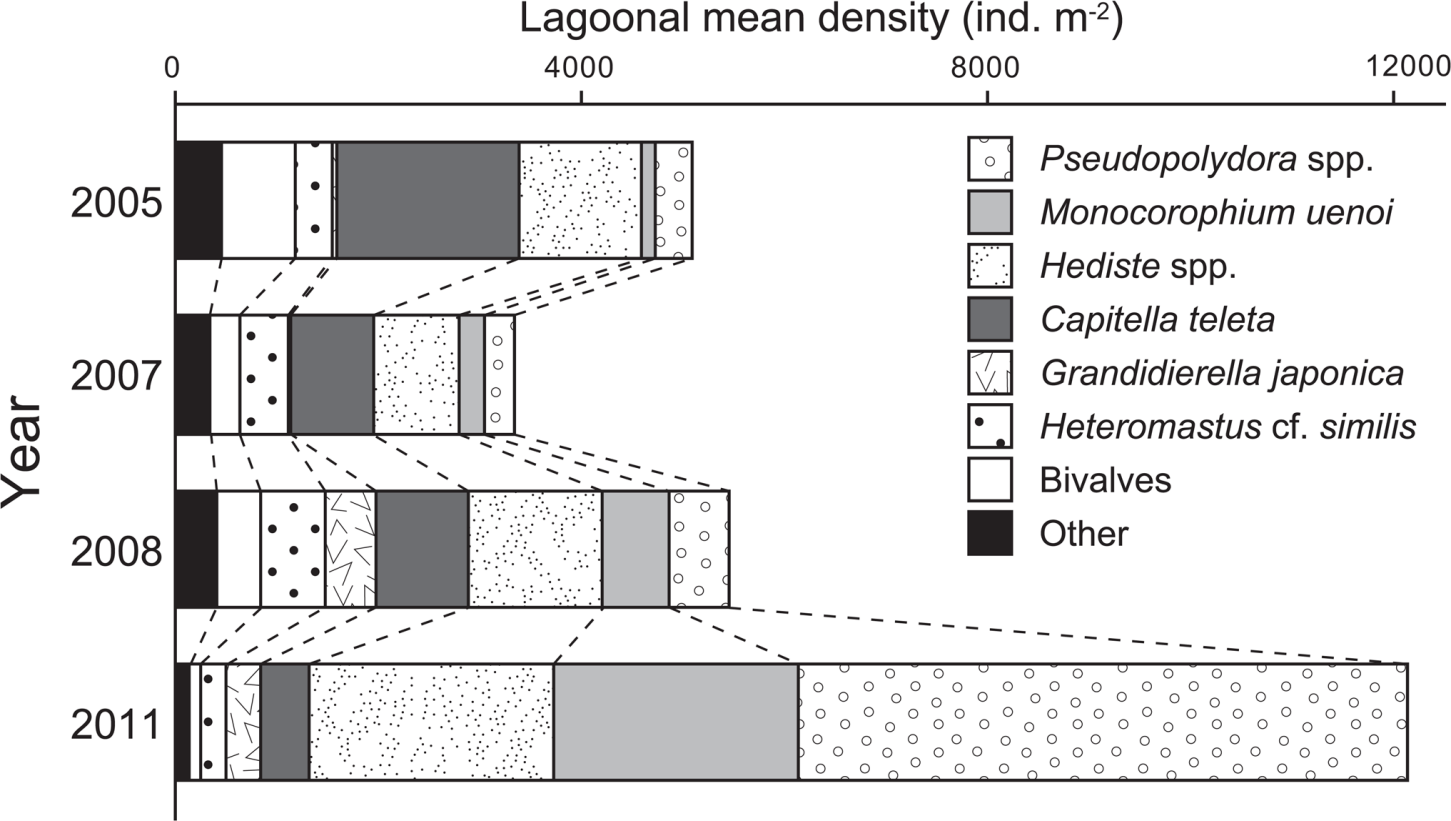
Interannual changes in community composition of macrozoobenthos. Data represent lagoonal mean density for each year (*n* = 30). Bivalves and other less dominant taxa were summated as “bivalves” and “others,” respectively.

The tsunami induced drastic changes in the composition of macrozoobenthos (Fig 8). In 2011, the community was characterized by a few dominant taxa including *Pseudopolydora* spp., *M*. *uenoi*, and *Hediste* spp., whereas other taxa (i.e., *C*. *teleta*, *H*. cf. *similis*, bivalves, and all other minor taxa) declined sharply. According to the SIMPER procedure (Table 3), the increased density of *Pseudopolydora* spp., *Hediste* spp., and *M*. *uenoi* and lower density of *C*. *teleta* in 2011 contributed most to the dissimilarity between 2011 and other years. The lower density of *H*. cf. *similis* in 2011 also contributed to the dissimilarity between 2011 and 2008.

**Table 3:** List of discriminant species that contributed most to the community difference between years before and after the tsunami.

Discriminant species ^a^	Mean density ^b^ (ind. m^–2^)		Cum. %
**2005 vs. 2011**	**2005**	**2011**	
*Pseudopolydora* spp.	379	5993	24.3
*Capitella teleta*	1789	476	37.4
*Hediste* spp.	1207	2401	50.4
*Monocorophium uenoi*	126	2414	61.2
**2007 vs. 2011**	**2007**	**2011**	
*Pseudopolydora* spp.	290	5993	29.1
*Hediste* spp.	853	2401	43.0
*M*. *uenoi*	254	2414	55.0
*C*. *teleta*	815	476	65.0
**2008 vs. 2011**	**2008**	**2011**	
*Pseudopolydora* spp.	552	5993	23.9
*Hediste* spp.	1544	2401	37.3
*M*. *uenoi*	605	2414	49.0
*C*. *teleta*	900	476	57.8
*Heteromastus* cf. *similis*	635	258	65.9

^a^ SIMPER procedure listed discriminant species until the cumulative contribution to inter-sample dissimilarity (Cum. %) achieved 60%.

^b^ Average density of discriminant species for each year was calculated.

## Discussion

The tsunami, with a 7.2-m inundation depth, caused extraordinary physical disturbances in Gamo Lagoon, leading to changes in sediment characteristics and macrozoobenthic abundance, diversity, and community structure at the whole-lagoon scale. The much larger R values for ANOSIM between 2011 and pre-tsunami years (2005 to 2008) suggest that tsunami-induced changes in macrozoobenthic community structure were extraordinal. The immediate impacts of the tsunami can be characterized as (1) creation of homogenous sand flats through removal of mud and deposition of drifting sea sand; (2) rapid population recovery of a few opportunistic taxa such as polychaetes and amphipods; and (3) loss of species richness, diversity, and evenness of the macrozoobenthic community.

Tsunami-induced changes in sediment granulometry had been reported from a shallow coastal bay and continental shelf [6, 9, 24]. At our study site, the most distinctive environmental modification was the disappearance of muddy sulfidic sediment. This was likely caused both by extraordinary shear stress under tsunami currents and massive sediment liquefaction during earthquake [25]. During the tsunami, a huge amount of sand deposits—mostly from beach and dune erosion—was settled onto the area <1 km from the coast line on the Sendai Plain [26]. These deposits led to the creation of the homogenous sandy bottom allover the lagoon. In Gamo Lagoon, contents of organic matters and sulfides sharply declined and the sediment became well oxidized after the tsunami (Eh ca. +300 mV; see also PCA plot in Fig 3). These indicated that the sediment environment was noticeably improved by the tsunami disturbance, i.e., recovery from sediment deterioration under organic pollution [27].

Before the tsunami, lagoon water contained high amount of phytoplankton (up to 100 μg L^-1^) that were the major source of SOM pool and supported the benthic food web (i.e., food for benthic invertebrates) [19, 20]. The significant increase in the SOM *δ* ^13^C value implied changes in the quality of the lagoonal SOM pool since *δ* ^13^C generally differed among source organic matters [20]. The lower SOM *δ* ^13^C value before tsunami (−22.7‰ on average) implies that the SOM mainly consisted of settling autochthonous phytoplankton (*δ* ^13^C; –23‰) [20]. In contrast, higher SOM *δ* ^13^C values in 2011 (−21.1‰) indicate the increasing contribution of ^13^C-enriched organic matter such as microphytobenthos on sediment surface (*δ* ^13^C; –20‰ to –10‰) [20]. These indicate that the tsunami modified both the quality and quantity of SOM stock in this lagoon, reflecting the washout of phytodetritus-rich mud and deposition of organic-poor sand.

In this lagoon, excess loading of phytodetritus caused the accumulation of organically enriched mud in inner portion [19]. Tsunami-induced changes in quality and quantity of SOM pool, as well as sharp reduction in mud content, suggest that the lagoon became less eutrophic, and thus, improving habitat quality for infaunal animals. After the tsunami, muddy sediment also disappeared in other lagoons along the Sendai Bay coast, including Idoura, Torinoumi, and Matsukawaura Lagoons ([15] and authors’ pers. obs.). Thus, replacement of muddy sediment with drifting sea sand should be one significant consequence of the 2011 tsunami common in all the estuarine lagoons along Sendai Bay.

Analyses based on historical data sets clearly demonstrated that species richness, diversity index (*H*ʹ), and evenness (*J*ʹ) sharply declined after the tsunami, although total macrozoobenthic density increased 2-fold. These changes were chiefly due to the disappearance of disturbance-sensitive taxa and the rapid recolonization and density increases of several opportunistic taxa after the tsunami disturbance. For example, dominant infaunal bivalves (*R*. *philippinarum*, *Macoma* spp., and *L*. *marilina*) and chironomid larvae were nearly extirpated after the tsunami. A qualitative survey conducted in 2011 [16, 17] also demonstrated that macroalgae-associated amphipods (*Ampithoe* sp., *Eogammarus possjeticus*, and *Melita* sp.), epifaunal gastropods (*Reticunassa festiva*, *Assiminea hiradoensis*, and *Cerithidea rhizophorarum*), epifaunal hermit crabs (*Pagurus minutus*), and deep-burrowing decapods (e.g., *Upogebia yokoyai* and *Nihonotrypaea japonica*) mostly disappeared after the tsunami. These suggest that tolerance of the tsunami disturbance and/or subsequent recovery potential differed among taxa even within the same taxonomic group.

Infaunal bivalves including *R*. *philippinarum*, *Macoma* spp., and *L*. *marilina* and deep-burrowing decapods seemed to be squeezed from the sediment under extensive liquefaction during the earthquake [25] and then washed away by the tsunami. Chironomidae sp. larvae would also have been washed away with resuspended mud because this taxa was specific to muddy habitats [21]. Other epifaunal and free-living species including epifaunal *R*. *festiva*, *A*. *hiradoensis*, *C*. *rhizophorarum*, and *P*. *minutus* and macroalgae-associated amphipods *Ampithoe* sp., *E*. *possjeticus*, and *Melita* sp. were also washed away with their substrates. In contrast, the bivalve *N*. *japonica* survived the tsunami, although the population size decreased. This species occurred abundantly in sandy unconsolidated sediments forced by high water currents [28]. In the Nanakita River estuary, this species was also highly abundant on unconsolidated sandy bottoms in the tidal channel and outside the levee [18, 19]. These imply that *N*. *japonica* is better adapted to unconsolidated habitat and may be more tolerant of physical disturbance compared to other sympatric bivalve taxa.

In Gamo Lagoon, macrozoobenthic community structure changed drastically after the tsunami, which was chiefly characterized by the density overshoots of *Pseudopolydora* spp., *Hediste* spp., and *M*. *uenoi*. One explanation for this is improving sediment characteristics. Before the tsunami, macrozoobenthos rarely inhabited the inner lagoon due to the accumulation of toxic H_2_S in sediment [19]. However, dominant polychaetes and amphipods formed dense colonies in the newly created sandy habitats throughout the lagoon. The higher intersample similarity of macrozoobenthic samples in 2011 (see nMDS plot in Fig 7) also showed that the community structure became spatially more homogenous, corresponding well to changes in sediment characteristics allover the lagoon.

Previous studies had shown that the dominant taxa in Gamo Lagoon preferred sandy oxidized sediments that do not contain toxic H_2_S, while only few species such as Chironomidae sp. were endemic to muddy sulfidic habitats [19, 29]. Our data showed that taxa preferring sandy habitats, such as *Hediste* spp., *Pseudopolydora* spp., and *M*. *uenoi*, significantly increased their population size, while Chironomidae sp. extirpated. These indicate that tsunami-induced changes in sediment characteristics (i.e., muddy sulfidic to sandy oxidized) were responsible for changes in abundance of dominant macrozoobenthos taxa. Therefore, improvement of sediment quality would be a significant consequence of the tsunami disturbance in this hypertrophic system.

Another explanation for the different speeds of population recovery among the dominant taxa were species-specific population turnover. Dominant taxa after the tsunami all seem to have opportunistic life-history traits (except for *Hediste diadroma* in the *Hediste* complex), allowing them to colonize rapidly and recover the population size within a relatively short period of time [30–33]. Breeding season of the univoltine polychaete *H*. *diadroma* is on spring tides in March to April [31], which was shortly after the 2011 tsunami. This would also allow the rapid recolonization of *H*. *diadroma*, as well as other opportunistic taxa, in our study site. In coastal soft-bottom habitats, increased density of opportunists was often found after a disturbance event such as an oil spill or hypoxia [4, 5, 33]. Present results highlight that the rapid recovery and proliferation of opportunists are the important characteristics of the soft-bottom community disturbed by the huge tsunami disaster. On the other hand, taxa with relatively low population turnover (e.g., bivalves) exhibited much slower population recovery, suggesting that population recovery rate after a huge pulsed disturbance depends closely to their life-history traits.

Huge tsunamis induced by earthquakes have occurred at intervals of 500 to 800 yr in northeastern Japan [12]. Thus, estuarine coastal habitats along the Pacific coast of Japan have been repeatedly affected by huge pulsed disturbances in the past. Our data demonstrate that the tsunami caused major habitat alterations in estuarine soft-bottom habitats through extensive sediment replacement. Therefore, it caused persistent changes in the habitat structure at a broader spatial scale, as well as having immediate impacts on local biotic communities. The rapid population recovery of opportunistic species would contribute to the functional recovery of estuarine soft-bottom communities shortly after the tsunami disturbance. On the other hand, delayed population recovery of specific functional groups (i.e., bivalves, macroalgae-associated amphipods, epifaunal gastropods, and deep-burrowing decapods) indicates the lacks in some ecosystem functions of the benthic community. Especially, disappearance of dominant infaunal bivalves with high biomass, such as *R*. *philippinarum* and *Macoma* spp., would induce a significant loss of ecosystem function since they play important roles in benthic ecosystem processes at the sediment-water interface, including bioturbation, metabolism, and nutrient regeneration [34, 35].

Our findings will help to improve our understanding of the impacts of a huge pulsed disturbance in estuarine and coastal communities. Present results emphasized both the vulnerability and resiliency of estuarine ecosystems in the face of extraordinal physical disturbance caused by a huge tsunami. At present, however, we do not have enough data to predict the succession pattern of the lagoon environment and associated biota in the future. Generally it takes several years or more to recover the benthic community structure after a intensive disturbance event [4, 6, 36]. Therefore, we will need to conduct further monitoring to know whether the lagoon environment is at a steady state and how long it will take to achieve ecological equilibrium in future.

## Supporting Information

S1 Data The excel file contains all data sets of sediment parameters and macrozoobenthos in Gamo Lagoon used in this manuscript. (XLS)
